# Factors Associated With Reduced Efficacy of Sitagliptin Therapy: Analysis of 93 Patients With Type 2 Diabetes Treated for 1.5 Years or Longer

**DOI:** 10.4021/jocmr1256w

**Published:** 2013-04-23

**Authors:** Akira Kanamori, Ikuro Matsuba

**Affiliations:** a Kanamori Diabetes Clinic, First floor, Prestige Sagami Yume Odori, 8-1-1 Sagamihara, Sagamihara City, Kanagawa 229-0031, Japan; b Matsuba Clinic, 2-159 Tsukagoshi, Saiwai-ku, Kawasaki City, Kanagawa 212-0024, Japan

**Keywords:** Sitagliptin, Type 2 diabetes, Hemoglobin A1c, Long-term administration

## Abstract

**Background:**

Several studies have shown the effectiveness of sitagliptin, a dipeptidyl peptidase-4 inhibitor, for type 2 diabetes, with a hypoglycemic effect being demonstrated both when it is administered alone or in combination with other oral antidiabetic agents. However, there are few reports about its long-term efficacy, although medications for diabetes need to be effective over the long term. This study (as part of ASSET-K) aimed to investigate the efficacy and safety of sitagliptin when it was administered for 1.5 years or longer, and to explore factors associated with reduction of the therapeutic response.

**Methods:**

Out of 375 patients treated with sitagliptin (50 mg/day) at Kanamori Diabetes Clinic between December 2009 and March 2012, 133 could be followed up for 72 weeks without interruption. After excluding 40 patients in whom the dosage and/or types of concomitant medications were modified during that period, the remaining 93 were included in this analysis. Clinical indices, such as blood glucose, HbA1c, and body weight, were investigated retrospectively. Compliance with diet and exercise therapy at 48 weeks was checked by a questionnaire.

**Results:**

In the 93 patients analyzed (sitagliptin monotherapy, n = 9; combination therapy, n = 77; and switching from an alpha-glucosidase inhibitor or glinide, n = 7), hemoglobin A1c (HbA1c) showed a significant decrease after 24 weeks (7.70 ± 0.73% at baseline vs. 6.90 ± 0.55% at 24 weeks), but then showed a slight increase at 48 weeks. HbA1c was subsequently maintained in the same range with no significant changes until 72 weeks. A positive correlation was noted between the changes of HbA1c and body weight from 24 to 48 weeks. Compliance with diet and exercise therapy was worse in patients showing a ≥ 0.3% increase of HbA1c (n = 37) from 24 to 48 weeks than in the others (n = 56). Multiple logistic regression analysis showed that both factors were independent determinants of the increase of HbA1c from 24 weeks onward.

**Conclusions:**

Sitagliptin showed good efficacy and safety when administered for 18 months as both monotherapy and combination therapy. Inadequate compliance with diet/exercise therapy and weight again may be associated with an increase of HbA1c over time during treatment with sitagliptin.

## Introduction

Sitagliptin is a dipeptidyl peptidase-4 inhibitor that is used to treat diabetes [1, 2], especially patients with type 2 diabetes and reduced incretin activity [3, 4]. When administered alone, it has no potential to cause hypoglycemia or weight gain. More than 2 years have passed since this drug became available clinically in Japan. Several clinical studies on the efficacy of sitagliptin have already been performed [5-10], and have clearly demonstrated that it exhibits a hypoglycemic effect when administered alone or in combination with other oral antidiabetic agents [11-15]. We are currently following 1,332 patients on sitagliptin therapy in the ASSET-K study [11, 12]. We performed the present analysis because hemoglobin A1c (HbA1c) tended to increase again after 1 year of sitagliptin therapy in the ASSET-K study. The follow-up period was only several months in most of the earlier studies and was a maximum of 1 year even in the clinical studies performed for development of this drug. However, because treatment of type 2 diabetes has to be continued over very long periods, such as 10 years or more, long-term efficacy and safety have to be confirmed for the drugs used to treat this disease.

Therefore, we conducted a retrospective analysis to investigate the long-term efficacy and safety of sitagliptin. We also aimed to clarify the reason for deterioration of HbA1c by comparing patients in whom HbA1c increased with those in whom a stable level was maintained.

## Materials and Methods

### Patients

Of the 375 patients treated with sitagliptin (50 mg/day) at Kanamori Internal Medicine Clinic between December 2009 and March 2012, 133 could be followed up continuously for 72 weeks. Among them, 40 patients were excluded from the study because the dosage or type of concomitant medications was changed during the follow-up period. Clinical indices, such as blood glucose, HbA1c, and weight, were investigated retrospectively in the 93 patients who could be followed up continuously for 72 weeks without any change in the dosage of sitagliptin or the dosages or types of concomitant medications. Patient compliance with diet/exercise therapy was investigated at 48 weeks by using a questionnaire [16].

### Ethical considerations

The present analysis was conducted as a part of the ASSET-K study. The ASSET-K protocol was devised in accordance with the Declaration of Helsinki and received ethical approval from the institutional review board of the Kanagawa Physicians Association. This study was undertaken in accordance with the Ethical Guidelines for Clinical Studies of the Japanese Ministry of Health, Labor, and Welfare.

### Statistical analysis

Results are given as the mean ± standard deviation. The profile of changes in each group was compared by the paired t-test, while the t-test and χ^2^ test were respectively used for comparing continuous variables and categorical variables between the groups. In all analyses, P < 0.05 was considered significant.

## Results

**Figure 1:**
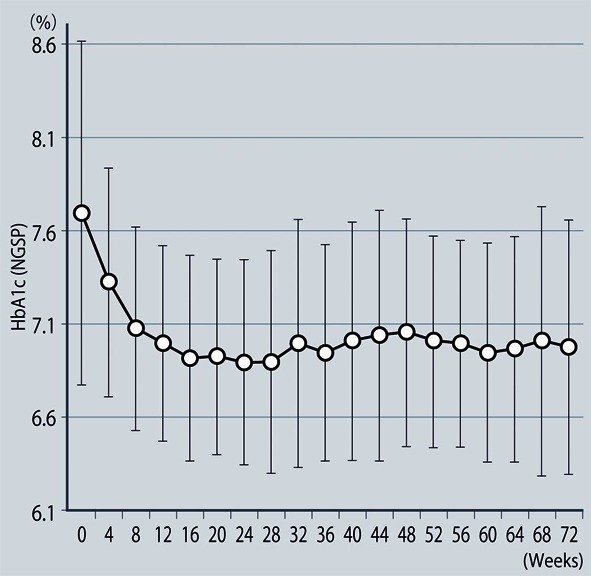
Changes of HbA1c (NGSP).

**Figure 2:**
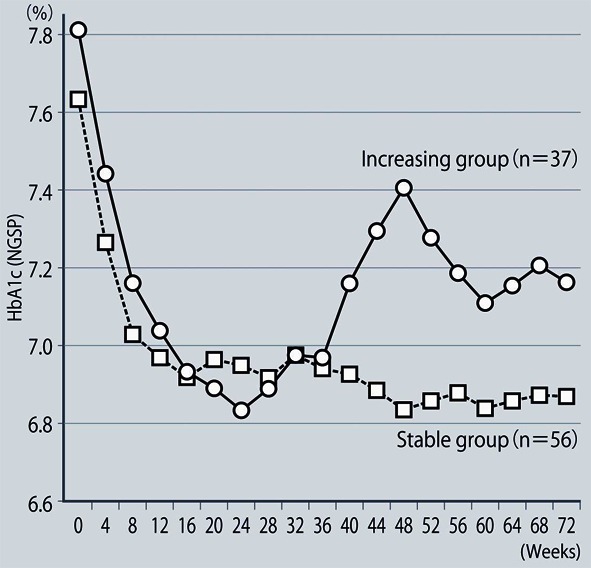
Comparison of groups with stable or increasing HbA1c levels.

**Figure 3:**
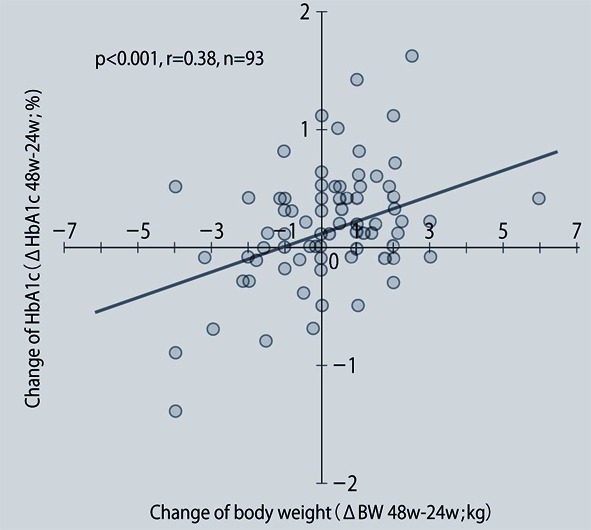
Relationship between the changes of body weight and HbA1c from 24 to 48 weeks after the start of sitagliptin therapy.

**Figure 4:**
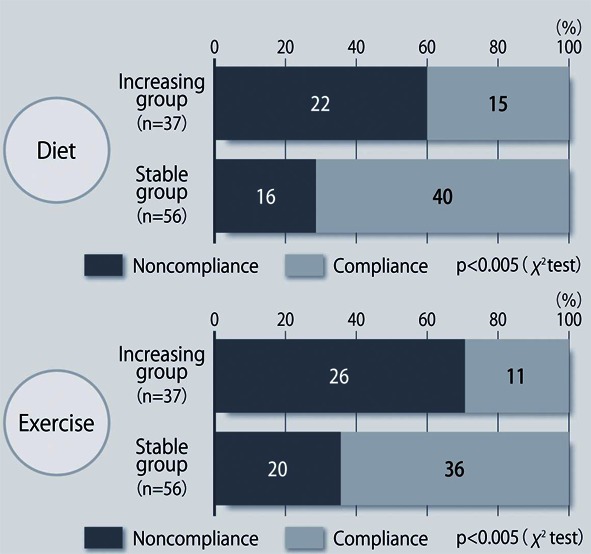
Compliance with diet/exercise therapy at 48 weeks after the start of sitagliptin treatment.

**Figure 5:**
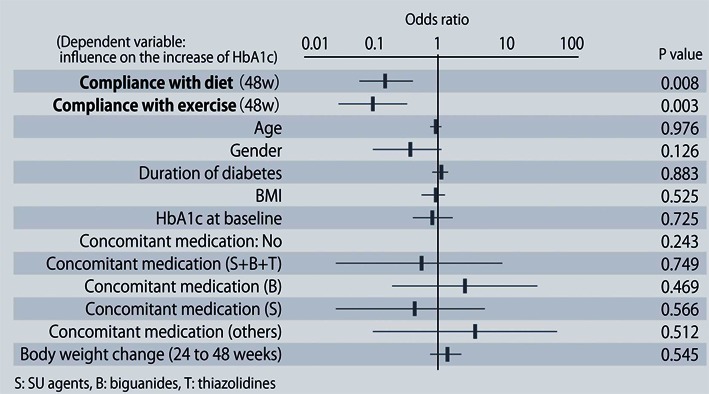
Factors related to a ≥ 0.3% increase of HbA1c from 24 to 48 weeks after starting sitagliptin treatment.

The demographic characteristics of the 93 patients were as follows. There were 52 men and 41 women with a mean (± SD) age of 63.6 ± 9.9 years. The mean duration of disease was 13.9 ± 6.3 years, mean body weight was 62.6 ± 11.9 kg, and mean HbA1c was 7.70 ± 0.73%. Sitagliptin was administered as monotherapy to 9 patients, or was combined with 1, 2, and 3 or more other antidiabetic drugs in 30, 33, and 14 patients, respectively. An alpha-glucosidase inhibitor or glinide was replaced by sitagliptin in 7 patients. HbA1c was 7.70 ± 0.73% at baseline and showed a significant decrease to 7.33 ± 0.61% after 4 weeks, 7.00 ± 0.52% after 12 weeks, and 6.90 ± 0.55% after 24 weeks of sitagliptin therapy (P < 0.001). It subsequently was maintained around that level with no significant change until 72 weeks. There was a slight increase from 24 to 48 weeks, but this was not statistically significant (Fig. 1). The profile of HbA1c up to 72 weeks was similar irrespective of the baseline level and was also similar in the sitagliptin monotherapy and combination therapy groups. Body weight showed no changes from baseline to 72 weeks. At 72 weeks, HbA1c was < 6.9% in 49 of the 93 patients (53%). Achievement of < 6.9% was more frequent when the baseline HbA1c was lower. HbA1c decreased gradually with time until 24 weeks, but showed different patterns of change from 24 weeks onward, namely, it did not increase and was stable in some patients, but it increased gradually with time from 24 weeks onward in others (Fig. 2). Clinical features were compared between the group with an increase of HbA1c by ≥ 0.3% and the group with an increase of < 0.3% between 24 and 48 weeks. As a result, none of the clinical indices that we assessed showed a significant difference between the two groups. However, body weight was significantly greater (P < 0.05) at 48 weeks than at 24 weeks in the increasing HbA1c group, while weight showed no significant change in the stable HbA1c group. A weak, but significant, positive correlation (P < 0.001, r = 0.34) was noted between the changes of HbA1c and body weight from 24 to 48 weeks, suggesting that an increase of HbA1c was associated with an increase of body weight (Fig. 3). At 48 weeks, compliance with diet and exercise therapy was investigated by a survey. As a result, the incidence of noncompliance was significantly higher in the increasing HbA1c group than in the stable HbA1c group (P < 0.005, χ^2^ test) (Fig. 4). Multiple logistic regression analysis was performed to identify factors related to a ≥ 0.3% increase of HbA1c from 24 to 48 weeks. As a result, noncompliance with diet and exercise therapy was found to be significantly related to the increase of HbA1c during that period (Fig. 5). No serious adverse reactions were noted during this study.

## Discussion

The present report describes the results obtained by follow-up of Japanese patients with type 2 diabetes for 72 weeks, during which period the dosages of sitagliptin and concomitant drugs for diabetes were not changed. It was confirmed that sitagliptin decreased that HbA1c level irrespective of the concomitant medications used, being effective for HbA1c, fasting plasma glucose, and postprandial glucose until 24 weeks of treatment. The rate of achieving an HbA1c < 6.9% (the target of treatment) was higher when the baseline HbA1c was lower.

It is often noted in clinical practice that HbA1c increases slightly if sitagliptin is used continuously for 6 months or more and body weight often increases in patients showing an increase of HbA1c. Inadequate compliance with diet and exercise therapy may be the cause of weight gain, but no studies have been done to confirm this. In the present study, we confirmed this for the first time, using data obtained by patient interview. In Japanese patients with type 2 diabetes, HbA1c has been reported to show seasonal fluctuation, usually decreasing from spring to summer and increasing from autumn to winter [17-20]. Because the patients enrolled in the present study started sitagliptin therapy from December to January, they reached autumn to winter after 44 weeks, when the risk of overeating increases in Japan due to Christmas parties, year-end parties, new year events, and new year parties. Because it is cold, patients probably tend to do less exercise than in summer, resulting in a lack of exercise. Thus, seasonal fluctuation was probably also related to the slight increase of HbA1c during sitagliptin therapy in the present study.

It is known that blood glucose improves until about 1 year after the start of treatment for diabetes, but subsequently increases with time over many years irrespective of the antidiabetic drugs used [21-23]. This phenomenon is described as the so-called secondary ineffectiveness of oral hypoglycemic agents. However, there was no likelihood of the increase of HbA1c in the present study being related to secondary ineffectiveness of sitagliptin [16].

In conclusion, the efficacy and safety of sitagliptin were maintained for 18 months as both monotherapy and combination therapy. In addition, inadequate compliance with diet/exercise regimens and weight gain may be related to the slight increase of HbA1c during long-term administration of sitagliptin.
